# A genome-wide association analysis of Framingham Heart Study longitudinal data using multivariate adaptive splines

**DOI:** 10.1186/1753-6561-3-s7-s119

**Published:** 2009-12-15

**Authors:** Wensheng Zhu, Kelly Cho, Xiang Chen, Meizhuo Zhang, Minghui Wang, Heping Zhang

**Affiliations:** 1 Department of Epidemiology and Public Health, Yale University School of Medicine, 60 College Street, New Haven, Connecticut 06520, USA

## Abstract

The Framingham Heart Study is a well known longitudinal cohort study. In recent years, the community-based Framingham Heart Study has embarked on genome-wide association studies. In this paper, we present a Framingham Heart Study genome-wide analysis for fasting triglycerides trait in the Genetic Analysis Workshop16 Problem 2 using multivariate adaptive splines for the analysis of longitudinal data (MASAL). With MASAL, we are able to perform analysis of genome-wide data with longitudinal phenotypes and covariates, making it possible to identify genes, gene-gene, and gene-environment (including time) interactions associated with the trait of interest. We conducted a permutation test to assess the associations between MASAL selected markers and triglycerides trait and report significant gene-gene and gene-environment interaction effects on the trait of interest.

## Background

Current advances in genotyping technologies, such as the Affymetrix 500 k GeneChip, make genome-wide association studies (GWAS) feasible for identifying common variants that underlie complex traits. Some of the recent genetic variants discovered by GWAS include age-related macular degeneration (AMD) [1,2], inflammatory bowel disease [3], and electrocardio-graphic QT interval [4]. Data from the 500 k genome-wide scan of the Framingham Heart Study (FHS) is available for use in the Genetic Analysis Workshop (GAW) 16. The FHS, a community-based cohort study initiated in 1948, aims to identify cardiovascular disease risk factors. FHS provides a collection of data from three generation families who had been followed up every 2 or 4 years over time. This longitudinal feature poses methodological challenges. Applying an efficient approach to analyzing the FHS longitudinal data may help in discovering new genetic variants in GWAS.

Previously, several approaches [5,6] were proposed to analyze the FHS 100 k data set; however, most of these did not directly deal with longitudinal data. These methods require the longitudinal measures to be summarized into one time-point trait by taking the average of several measures or by using the family-based association test (FBAT) principal-components method [6]. It is inevitable that there may be some loss of information by using the summary trait values [7]. Furthermore, when applying the adjustment of FBAT-principal-components method in GWAS, it is difficult to include environment factors such as sex and age.

In our study, we use the multivariate adaptive splines for analysis of longitudinal data (MASAL) presented by Zhang [8] to analyze the FHS longitudinal data. MASAL is a nonparametric regression approach that was developed specifically to handle longitudinal data. MASAL not only accommodates time-varying covariates, but also allows interactions between gene and environmental factors and between time and covariates [9]. Here we demonstrate and apply MASAL to identify genes, gene-gene, and gene-environment interactions in relation to the trait triglyceride (TG) level in GWAS using FHS data in GAW16 Problem 2.

## Methods

### MASAL

We present a brief review of MASAL and refer to Zhang [8,10] for the details. Let *y*_*ij*_, *t*_*ij*_, and *x*_*k*, *ij *_denote the response variable, time-dependent covariate, and *k*^th ^non-time-dependent-covariates (including both genetic and environmental covariates) for the *i*^th ^subject at the *j*^th ^exam, where *j *= 1, ..., *T*, *i *= 1, ..., *n*, *k *= 1, ..., *p*; *n *is the number of study subjects and *T*_*i *_is the number of exams for the *i*^th ^subject. In MASAL, we consider the following nonparametric model:

where *f *is an unknown smooth function and *ε*_*ij *_is the error term.

Based on a set of observations, MASAL selects a model using a forward step from the following class of functions:

where *β*_*m *_is the regression coefficient and *B*_*m*_(**x**) is a special basis function of the *p *+ 1 covariates **x **= (*x*_1_, ..., *x*_*p*+1_) (*m *= 1, ..., *M*), and *M *is the number of terms. Specifically, *B*_*m*_(**x**) is either one of (*x*_*k *_- *τ*)^+ ^and *x*_*k *_or their product (*k *= 1, ..., *p *+ 1), and *a*^+ ^= max(*a*,0) for any number *a *and *τ *is called a knot.

In the forward step, terms are added to minimize the (weighted) sum of squared residuals: , where  and  is the predicted value of *y*_*i*_, and **W**_*i *_is the within-subject covariance matrix for , *i *= 1, ..., *n*. After the forward step, all knots are found and each corresponding basis function will be treated as if it is a given predictor. In the backward step, based on generalized cross-validation (GCV), we delete one least significant term from the large model at a time. The final model we select is the one that yields the smallest , where *WLS*_*l *_is the *WLS *of a reduced model with *l *terms and *λ *(usually *λ *= 4 [10,11]) is the penalizing parameter for model complexity.

### GWA analyses with MASAL

In GWAS, we use MASAL to establish the relationship between a trait of interest and genomic markers as well as other non-genetic covariates. MASAL starts with a model that contains only the intercept a *α*, and it grows the model by adding terms that minimize the *WLS *in the forward step, and then it selects a final model by deleting one least significant term at a time in the backward step. In general, the final MASAL model is

where  represents terms containing any genetic component (i.e., single-nucleotide polymorphism (SNP), SNP-SNP interaction, or SNP-covariate interaction),  refers to non-genetic covariate terms, and ,  is the estimate of the corresponding regression coefficients.

To access associations between the selected SNPs and the trait of interest, we define a Wald statistic to test whether ***β ***= 0, where ***β ***= (*β*_1_, ..., *β*_*k*_)'. The Wald statistic can be written as

where , and  is the estimated covariance matrix of . We use a permutation procedure to establish the null distribution of *W *The permutation test is done by randomly assigning the phenotype while keeping the set of genotypes intact for each individual and then performing the GWA analysis using MASAL. It is noteworthy that non-genetic covariates go together with the phenotype.

### Study design

We perform GWA analyses of TG trait with MASAL. We consider the genotype at every SNP as a covariate in the model in addition to sex and age variables. MASAL has the option of setting the maximum order of interactions in the model. We set it to three in our analyses because it is difficult to interpret interactions higher than the third order. We first use MASAL to perform GWA analyses in the Offspring Cohort, in which the repeated TG values and the familial correlations are properly accounted for in the analysis. Next, we perform GWA analyses with MASAL in the Original Cohort in which the subjects are considered to be independent, whereas the longitudinal trait values are considered to be correlated. Then, we examine significant SNPs, SNP-SNP, and SNP-covariate interactions in the two generation data sets analyzed and compare the level of concordance of significant associations in the two samples.

In the Offspring Cohort, some pedigrees have more than 100 subjects, which cannot be treated as independent individuals. These families with repeated traits induce a large covariance matrix in MASAL and thus markedly limit its efficiency. Therefore, in the analyses of Offspring Cohort, we split all pedigrees into sibship units according to the information from the Original Cohort. We obtained 1,767 sibship units, for which each sibship unit consists of one set of siblings and their spouses from each nuclear family. All subjects included in our study have all TG trait values (Exams 1, 3, 5, and 7) and genotypes. In the Original Cohort, we used 146 individuals who have all TG trait values (Exams 7 and 11) and genotypes. All of these subjects were genotyped for 488,146 SNPs.

## Results and discussion

We applied MASAL to analyze the FHS 500 k SNP data set (GAW 16 Problem 2). Before the analysis, TG level values were log-transformed to approximate a normal distribution, although there is no such limitation when using MASAL. Furthermore, in order to minimize false-positive associations due to rarer SNPs and genotyping artifact, we limited our analyses to SNPs with minor allele frequency ≥ 10% and the *p*-value for testing Hardy-Weinberg equilibrium <0.001. Thus, there were a total of 294,434 SNPs remaining in our analysis.

In the analyses of Offspring Cohort, the fitted model given by MASAL is

where *t *is the age of exam, *s *is the indicator for sex, and  is the genotype of the SNP rs number in the *c*^th ^chromosome. The value of corresponding Wald statistic is 325.73. Similarly, for the analyses of original cohort, the fitted model given by MASAL is

and the value of the corresponding Wald statistic is 359.575.

**Figure 1:**
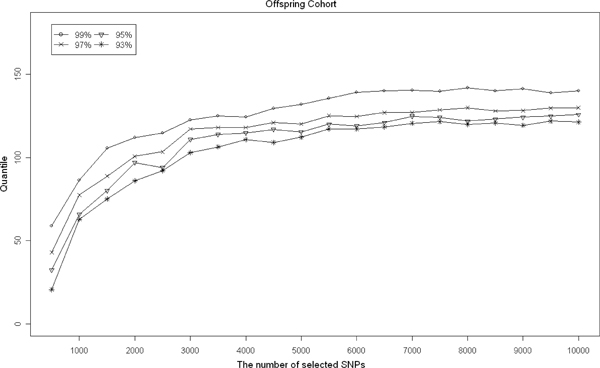
**The trends of the quantiles (93%, 95%, 97%, and 99%) with the increase of the number of selected SNPs based on the Offspring Cohort**. The vertical axis represents the value of the quantile and the horizontal axis represents the number of selected SNPs in each permutation study.

We attempted to establish the null distributions of the two Wald statistics by using a permutation procedure and calculating the *p*-values of the two tests. However, the permutation study based on the entire genome is extremely time-consuming. Thus, instead of using the entire genome, we randomly selected a series of subsets of SNPs in 500 increments (e.g., 500, 1000, 1500, ..., 10,000 SNPs) of 500 k SNPs to characterize the pattern of the empirical distribution of *W*. Based on each subset, we used the above permutation procedure to establish the empirical distribution of *W*and calculated 93%, 95%, 97%, and 99% quantiles. In Figure 1, we illustrate the trends of the four quantiles showing the increase in the number of selected SNPs for the Offspring Cohort. The trends of these quantiles for the Original Cohort are similar, so we omitted them in Figure 1 due to the limited space. These results indicate a convergence when the number of selected SNPs is more than 7,000. As a result, we established the null distributions of *W *based on 10,000 randomly selected SNPs.

The adjusted *p*-value of the Offspring Cohort based association test is less than 0.001, which suggests strong associations between the SNPs selected by the final model and TG trait. In contrast, the adjusted *p*-value of the Original Cohort based association test is 0.056, which indicates marginally significant associations between the selected SNPs and TG trait. Our results confirm that MASAL can properly take account of the familial correlations in the GWA analysis. MASAL identified 13 significant SNPs for the Offspring Cohort and 6 SNPs for the Original Cohort. Table 1 lists the significant SNPs selected by MASAL, their chromosomal positions, and the nearest gene(s). Although significant associations identified in the two samples do not overlap, the two fitted models exhibit SNP-SNP and SNP-environment interactions. It is not entirely surprising that there is no overlap among the significant SNPs between the two cohorts because for example, the characteristics such as age in the two cohorts are different. It is known that there is a steady increase of TG levels with age [12]. Furthermore, the two MASAL models include the age or SNP-age interaction terms, confirming the age effect. Kooner et al. [12] reported an association between *MLXIPL *on chromosome 7 and TG; and Kathiresan et al. [13] found five loci on chromosomes 1, 7 (*TBL2 *and *MLXIPL*) 8, and 19 to be associated with TG. Our models do not include SNPs in those regions. Further investigation is warranted to confirm our findings. Although MASAL is a unique approach to accommodating correlated phenotypes in genetic studies, its potential has not been fully explored. It is beyond the scope of this short article to compare MASAL with other methods, but it is a highly worthy effort to thorough study the utility of MASAL for longitudinal genetic data.

**Table 1:** Significant SNPs selected by MASAL

Data set	SNP	Locus	Nearest gene(s)
Offspring Cohort	rs4367528	8q12	*RLBP1L1*
	rs16860145	3q13.2	*CD200R2*
	rs4074863	10q26	*FLJ46300, TCERG1L*
	rs9828013	3p25	*WNT7A*
	rs41442345	4q23	*BANK1*
	rs11150610	16p11.2	*ITGAM*
	rs5015152	3q26.3	*NLGN1*
	rs17117113	5q33	*KIF4B*
	rs1361536	9q21	*KRT18P24, CHCHD9*
	rs17630545	8q23	*CSMD3*
	rs7204454	16q23	*CDH13*
	rs1321130	1q42	*FAM89A, FLJ30430*
	rs2514930	11q21	*NAALAD2*
Original Cohort	rs6835031	4q22	*TIGD2*
	rs4984982	16p13.3	*LMF1*
	rs16995794	20q13.2	*RPSAP1*
	rs17783132	14q24	*BATF*
	rs11688196	2p23	*TRNAL-AAG*
	rs9643584	8q13	*CPA6*

## Conclusion

In this report, we proposed a testing procedure to perform GWAS for longitudinal data, using a nonparametric regression method (MASAL) presented by Zhang [8]. In contrast to other GWA methods, our testing procedure has two novel features. First, it can handle longitudinal data without combining longitudinal measures into a one-time-point measure in GWAS. Second, it can accommodate gene-gene, gene-environment, and time-covariate interactions in GWAS. Using MASAL, we analyzed the FHS 500 k genotype data (GAW 16 Problem 2) using TG as the trait of interest and found some significant gene-gene and gene-environment interaction effects on TG trait. These results indicated that MASAL is useful for exploring gene-gene and gene-environment interactions in the GWAS of longitudinal data.

We used a permutation procedure to establish the null distribution of the Wald statistic and then estimated the significance level. However, the computation time was lengthy, especially for the large pedigree and large number of exams of each subject. Theoretical studies exploring the asymptotic distribution of the involved statistic would be useful.

## List of abbreviations used

AMD: Age-related macular degeneration; FBAT: Family-based association test; FHS: Framingham Heart Study; GAW: Genetic Analysis Workshop; GCV: Generalized cross-validation; GWAS: Genome-wide association studies; MASAL: Multivariate adaptive splines for the analysis of longitudinal data; SNP: Single-nucleotide polymorphism; TG: Triglyceride.

## Competing interests

The authors declare that they have no competing interests.

## Authors' contributions

WZ and HZ designed the study and carried out the data analysis. WZ, KC, and HZ drafted the manuscript. KC, XC, MZ, and MW participated in data analysis.

All authors read and approved the final manuscript.
